# Supplementary figures and images for: Genome-wide association study reveals genetic basis and candidate genes for chlorophyll content of leaves in maize (Zea mays L.)
Source: PeerJ. 2024 Oct 7;12:e18278. doi: 10.7717/peerj.18278 (PMC11466220; doi:10.7717/peerj.18278)

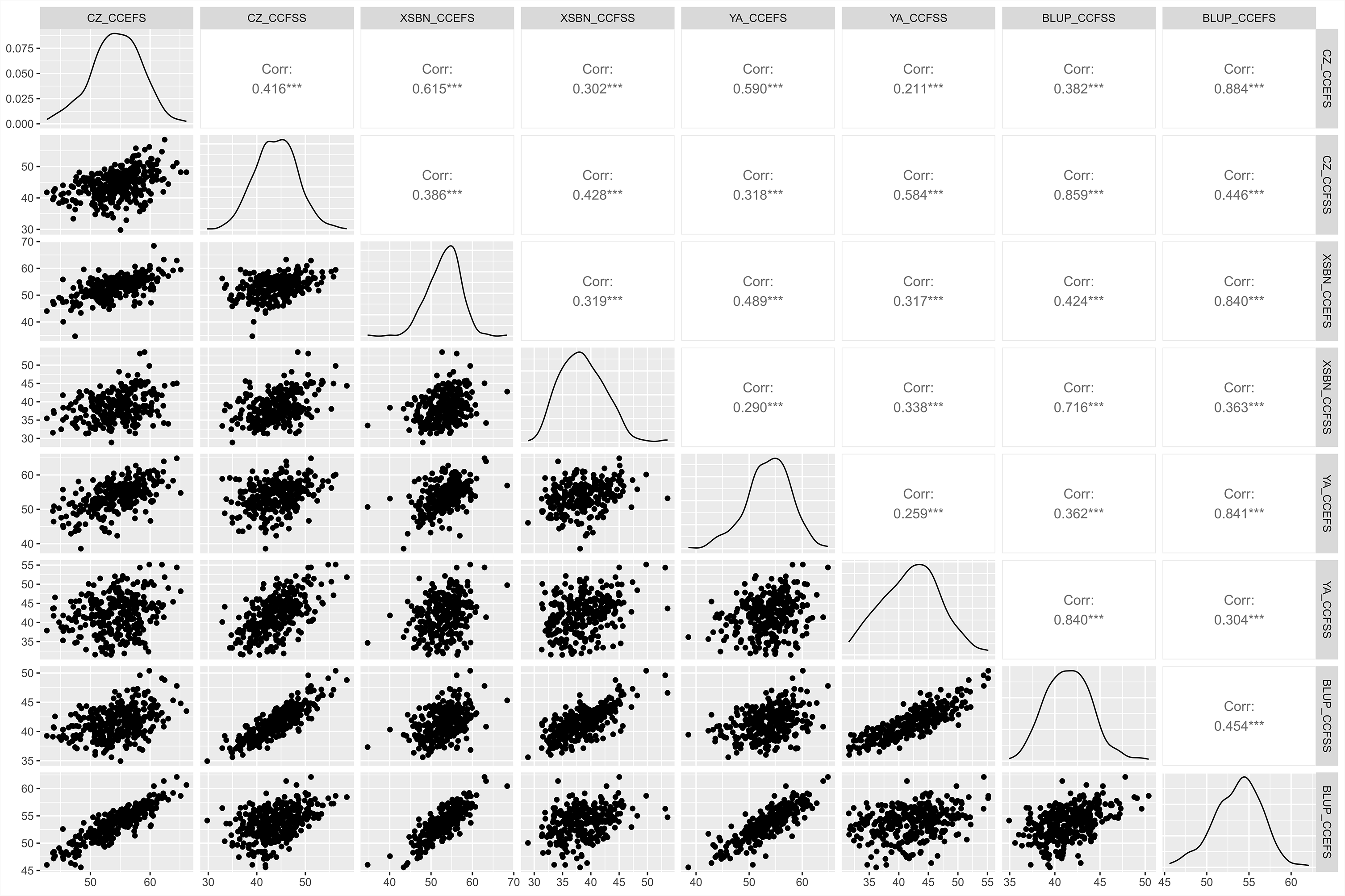

Supplement: Supplemental Information 1 — Figure S1: Correlation analysis for the CCFSS and CCEFS of maize lines in different environments. *** represents the significant correlation with p-value < 0.001. CCFSS and CCEFS represent chlorophyll contents of fifth leaves at seedling stage and ear leaves at filling stage, respectively.; Figure S2: Heat map of the expression patterns of 177 candidate genes. The value used in the figure is the log10(Exp +1) conversion ratio of the counts of standardized PRKM in different maize tissues in various development stages. Columns and rows are clustered according to expression similarity. Compared with different periods of a specific gene, blue, yellow, and red colors represent higher, moderate, and lower expression, respectively. Table S1: Information of 334 maize lines. Table S2: Significantly associated SNPs detected by the MLM model; Table S3: Candidate genes for 15 CC-related SNPs; Table S4: Top 20 gene ontology terms; Table S5: Top 20 KEGG pathways; Table S6: Variations of eight hub candidate genes; Table S7: Significant markers identified in gene-based association analysis; Table S8: Expression levels of eight hub candidate genes. [file peerj-12-18278-s001.zip › Supplementary Files/Figure S1.tif]

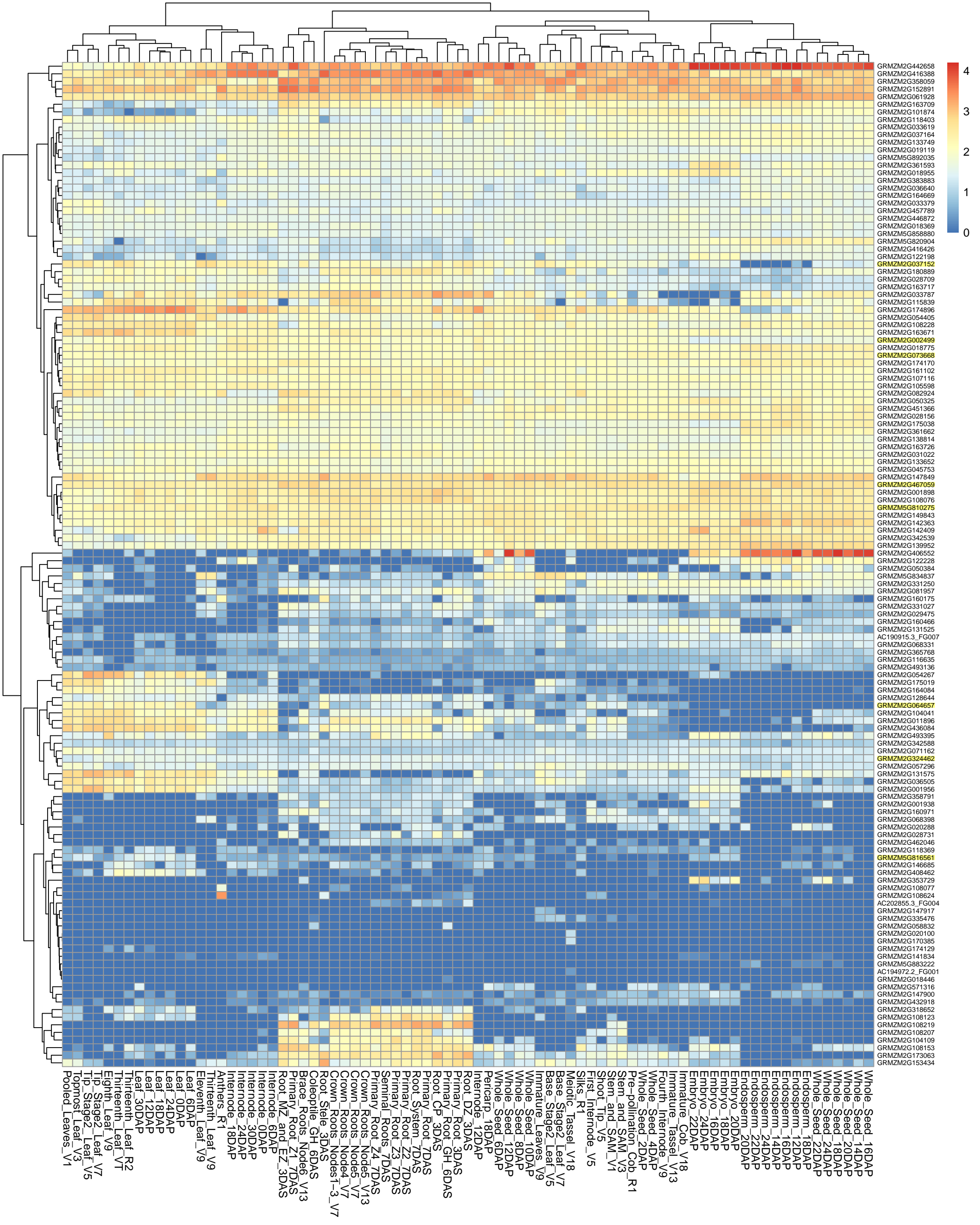

Supplement: Supplemental Information 1 — Figure S1: Correlation analysis for the CCFSS and CCEFS of maize lines in different environments. *** represents the significant correlation with p-value < 0.001. CCFSS and CCEFS represent chlorophyll contents of fifth leaves at seedling stage and ear leaves at filling stage, respectively.; Figure S2: Heat map of the expression patterns of 177 candidate genes. The value used in the figure is the log10(Exp +1) conversion ratio of the counts of standardized PRKM in different maize tissues in various development stages. Columns and rows are clustered according to expression similarity. Compared with different periods of a specific gene, blue, yellow, and red colors represent higher, moderate, and lower expression, respectively. Table S1: Information of 334 maize lines. Table S2: Significantly associated SNPs detected by the MLM model; Table S3: Candidate genes for 15 CC-related SNPs; Table S4: Top 20 gene ontology terms; Table S5: Top 20 KEGG pathways; Table S6: Variations of eight hub candidate genes; Table S7: Significant markers identified in gene-based association analysis; Table S8: Expression levels of eight hub candidate genes. [file peerj-12-18278-s001.zip › Supplementary Files/Figure S2.pdf]
